# Parametric cardiovascular magnetic resonance imaging in takotsubo syndrome: a case report

**DOI:** 10.1093/ehjcr/ytae016

**Published:** 2024-01-08

**Authors:** Ritesh Sunnasy, Raad Hashem Mohiaddin

**Affiliations:** Royal Brompton and Harefield Hospitals, Guy’s and St Thomas’ NHS Foundation Trust, Sydney Street, SW3 6NP London, UK; Royal Brompton and Harefield Hospitals, Guy’s and St Thomas’ NHS Foundation Trust, Sydney Street, SW3 6NP London, UK; National Heart and Lung Institute, Imperial College London, London, UK

**Keywords:** Cardiovascular magnetic resonance, Takotsubo syndrome, Cardiac imaging, Parametric mapping, Late gadolinium enhancement, Case report

## Abstract

**Background:**

Takotsubo syndrome (TTS) causes angina with ventricular dysfunction that can mimic acute coronary syndrome. Normal coronary angiography leads to cardiovascular magnetic resonance imaging (CMR) in the diagnostic pathway. Historically, TTS was thought to be associated with the absence of late gadolinium enhancement on CMR. This case report highlights the presence of late gadolinium enhancement in a case of TTS while demonstrating the other characteristic findings, including quantitative parametric T_1_/T_2_ mapping.

**Case summary:**

A 69-year-old lady was admitted with chest pain and shortness of breath. She was found to have classical TTS with the characteristic wall motion abnormalities on echocardiogram, left ventricular angiogram, and CMR. Her CMR also demonstrated strongly positive myocardial T_1_/T_2_ mapping that matched the wall motion abnormalities and the less frequently described positive early and late gadolinium enhancement.

**Discussion:**

This case highlights the diagnostic pathway in TTS and the ability of CMR to make a diagnosis in myocardial infarction with non-obstructed coronary arteries. We describe the characteristic cardiac imaging features of TTS while discussing the positive late gadolinium enhancement patterns that may help confirm the diagnosis.

**Learning points**

- Cardiovascular magnetic resonance imaging is the key diagnostic imaging modality in myocardial infarction with non-obstructed coronary arteries particularly when performed early.
- Cardiovascular magnetic resonance imaging can identify less frequently appreciated late gadolinium enhancement patterns that may help confirm the diagnosis of takotsubo syndrome.

## Introduction

Takotsubo syndrome (TTS) is a well-recognized cause of acute chest pain. With anginal symptoms, electrocardiogram (ECG) abnormalities, raised serum troponin, and wall motion abnormalities on echocardiography, patients are often promptly investigated and managed as presumed acute coronary syndrome (ACS). Unobstructed coronary arteries on invasive angiography result in a change in the working diagnosis from ACS to myocardial infarction with non-obstructed coronary arteries (MINOCA). Myocardial infarction with non-obstructed coronary arteries represents a provisional diagnosis encompassing a diverse range of causes including TTS, myocarditis, coronary spasm, and cardiomyopathies. Cardiovascular magnetic resonance imaging (CMR) is the key diagnostic investigation after angiography to identify the exact aetiology of MINOCA and thereby help guide management.^1^ We present a case of classical TTS with characteristic imaging features and with less frequently described late gadolinium enhancement (LGE).

## Summary figure

**Figure:**
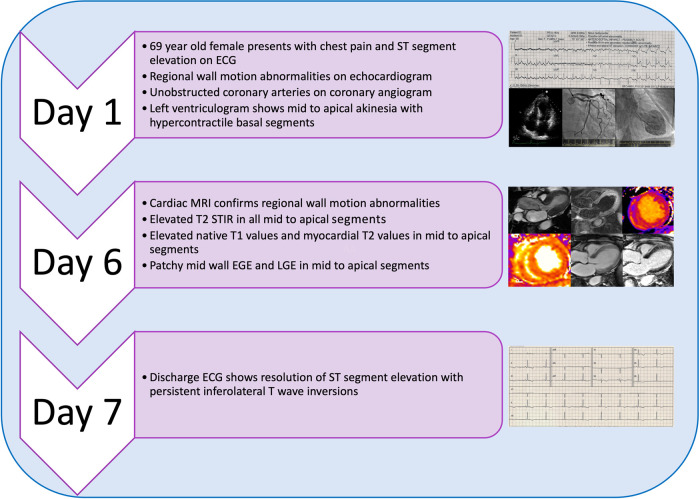


## Case presentation

A 69-year-old female called an ambulance after experiencing chest pain and breathlessness. Her past medical history included chronic obstructive pulmonary disease, hypercholesterolaemia, hypothyroidism, and being an ex-smoker of 16 years.

**Figure 1:**
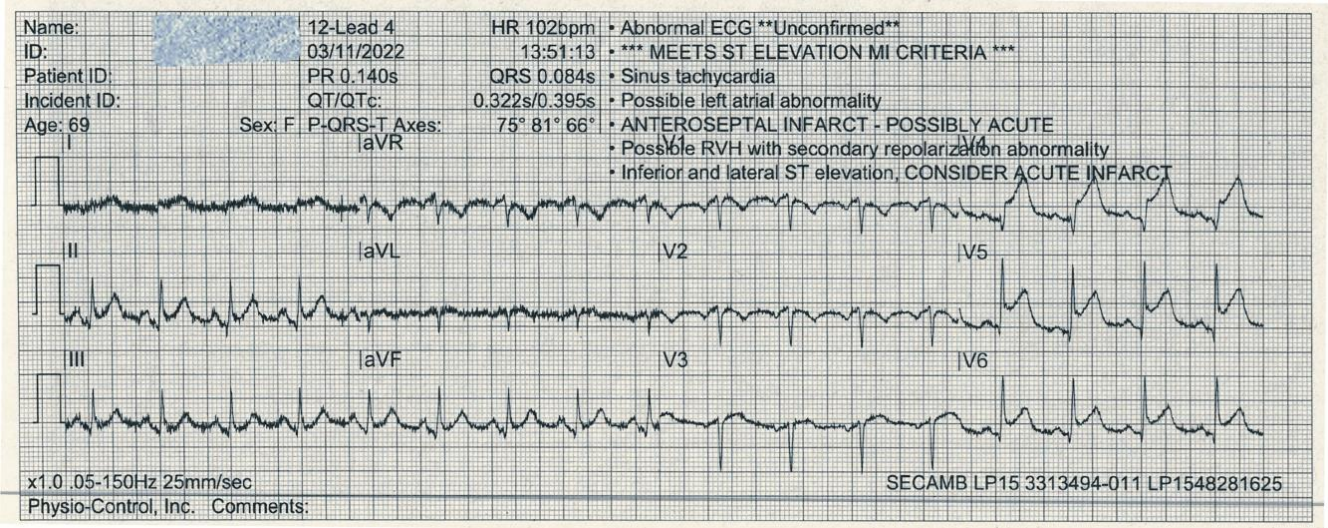
Initial electrocardiogram. Ambulance electrocardiogram demonstrating sinus tachycardia with inferolateral ST elevation.

On attendance, paramedics found her pale and tachypnoeic. She had a respiratory rate of 30 b.p.m. with bilateral basal crackles, oxygen saturation of 89% on room air, a heart rate of 102 b.p.m., and blood pressure of 109/63 mmHg and was febrile with a temperature of 38.4°C. Her jugular venous pressure was not significantly elevated, heart sounds were normal with no added murmurs, and she did not have any peripheral oedema. Electrocardiogram showed sinus tachycardia with inferolateral ST elevation (*Figure 1*).

The most significant differential diagnosis from her presentation was acute ST elevation myocardial infarction (STEMI). She was therefore given antiplatelet loading therapy (aspirin 300 mg and ticagrelor 180 mg) and transported to the nearest primary percutaneous coronary intervention (PPCI) centre. Differential diagnoses include pericarditis, myocarditis, acute aortic syndrome, or TTS.

At the PPCI centre, blood tests showed a markedly elevated high-sensitivity cardiac troponin I at 29 421 ng/L (normal range 0–16 ng/L). Echocardiography demonstrated severely impaired left ventricular function with ejection fraction visually estimated to be 30–35%, with akinesia of mid to apical left ventricular segments and hypercontractile basal segments (*Video 1*; see Supplementary material online, *Videos S1*–*S3*).

Urgent invasive coronary angiogram revealed unobstructed coronary arteries with mild irregularities (see Supplementary material online, *Videos S4*–*S9*). Left ventriculogram identified once again an akinetic mid to apical left ventricle with hypercontractile neck (*Video 2*).

Myocardial infarction with non-obstructed coronary arteries was therefore the working diagnosis for her presentation, and she was referred for an inpatient CMR scan to identify the precise aetiology of her MINOCA, which she underwent 5 days later. This showed a similar pattern of wall motion abnormalities to the left ventriculogram (*Video 3A–C*) typical of classical pattern TTS. Left ventricular ejection fraction from short-axis volumetric quantification was borderline impaired for her age at 60% (normal range 60–78%^2^). Right ventricular function was normal with an ejection fraction of 70% (normal range 57–81%^3^).

**Figure 2:**
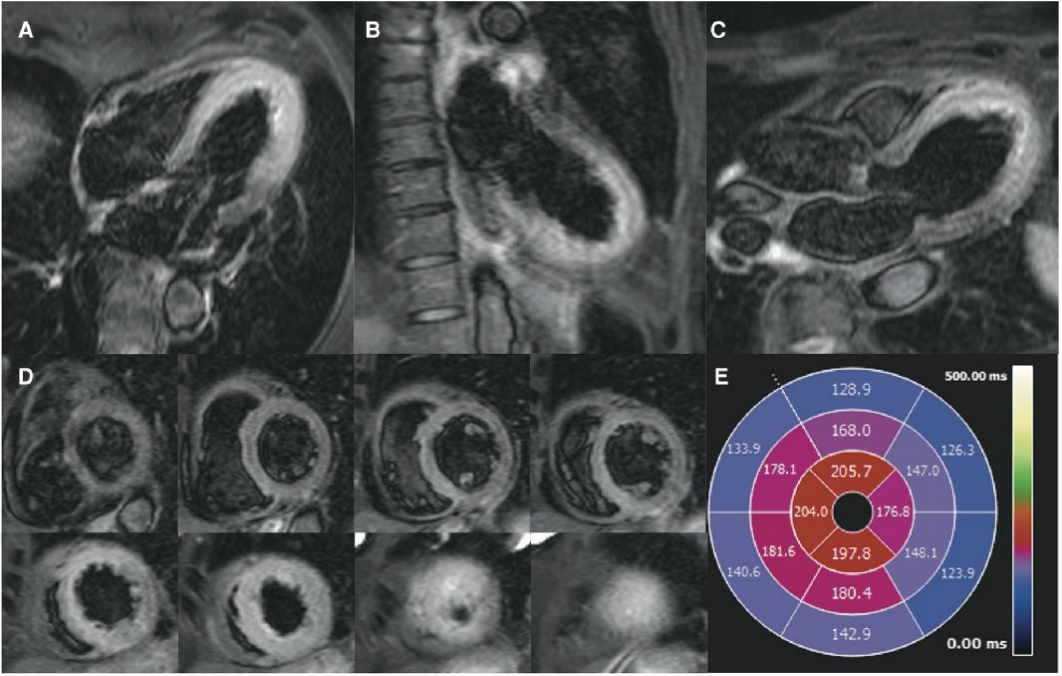
T_2_ short tau inversion recovery. Horizontal long axis (*A*), vertical long axis (*B*), left ventricular outflow tract view (*C*), short axis (*D*), and quantitative polar map (*E*).

T_2_ short tau inversion recovery (STIR) sequences demonstrated hyperintense myocardial signal intensity in the mid to apical regions (*Figure 2*).

**Figure 3:**
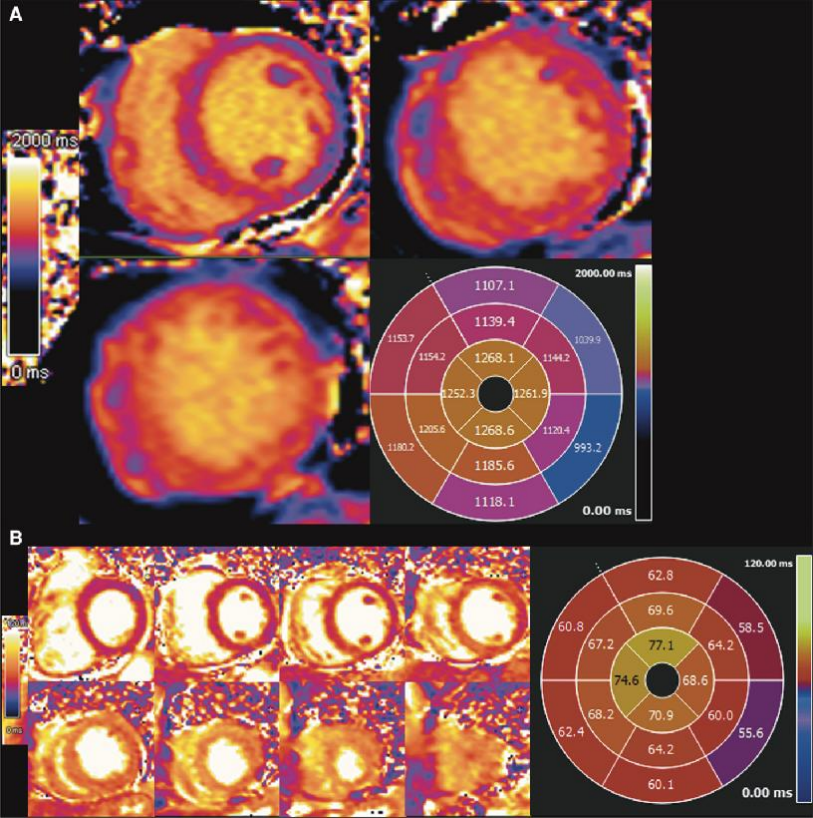
(*A*) Colour depiction of T_1_ mapping of three short-axis slices with quantitative polar map. (*B*) Colour depiction of T_2_ mapping from base to apex short axis with quantitative polar map.

Native T_1_ mapping (*Figure 3A*) and T_2_ mapping sequences (*Figure 3B*) demonstrated increased relaxation times throughout the mid to apical regions reflecting oedema (normal native T_1_ range 975–1065 ms; normal T_2_ < 55 ms for 1.5 T Siemens MAGNETOM Sola MRI scanner).

**Figure 4:**
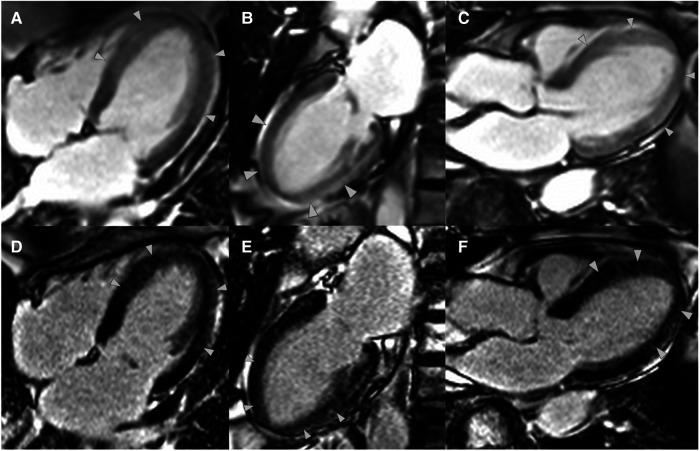
Early gadolinium enhancement in the horizontal long-axis (*A*), vertical long-axis (*B*), and left ventricular outflow tract (*C*) views. Late gadolinium enhancement in the horizontal long-axis (*D*), vertical long-axis (*E*), and left ventricular outflow tract (*F*) views. Arrowheads indicate areas of increased signal intensity.

**Figure 5:**
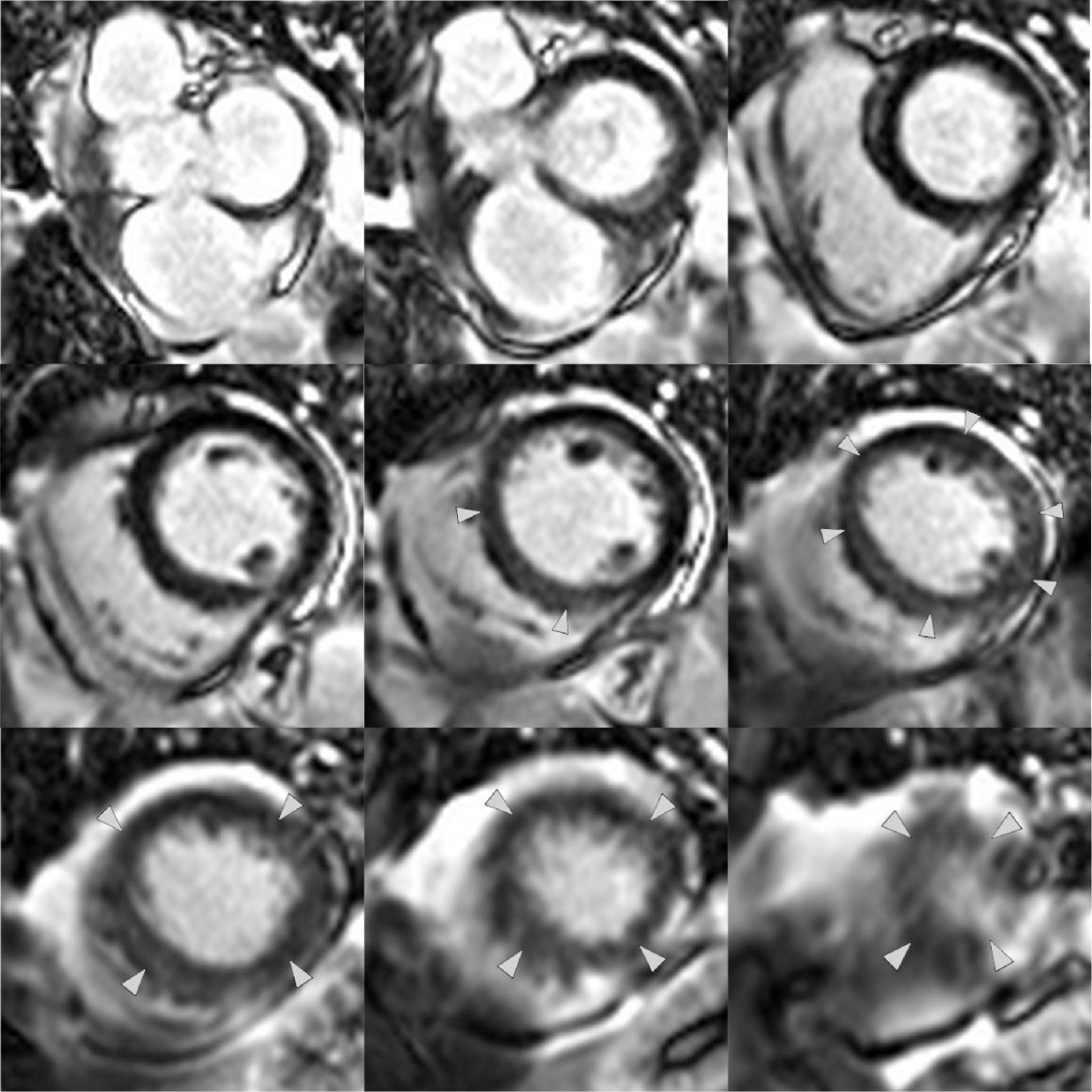
Late gadolinium enhancement in the short-axis views from base to apex. Arrowheads indicate patchy/mid-wall diffuse enhancement.

Early gadolinium sequences (*Figure 4A–C*) and late gadolinium sequences (*Figures 4D–F* and *5*) demonstrated subtle diffuse and patchy/diffuse mid-wall enhancement respectively in mid to apical segments. No thrombus was identified. Absence of subendocardial fibrosis ruled out myocardial infarction, and absence of subepicardial fibrosis made myocarditis less likely.

Antiplatelet medications were discontinued, and she was commenced on heart failure medication (ramipril 1.25 mg twice a day, dapagliflozin 10 mg once a day, and eplerenone 25 mg once a day). She remained haemodynamically stable and was subsequently discharged with resolving ST-segment elevation but with persistent inferolateral T-wave inversions (see Supplementary material online, *Figure S1*). A 6-week follow-up echocardiogram was arranged.

## Discussion

Here, we describe a case of classical TTS illustrated by characteristic CMR cine sequences, T_2_ STIR, T_1_ and T_2_ mapping, and less frequently described positive early gadolinium enhancement (EGE) and LGE findings. The typical patient with TTS is a post-menopausal woman presenting with acute angina and ECG findings activating the ACS treatment pathway.^4^ Cardiac imaging, with echocardiography as first line,^1^ identifies wall motion abnormalities that extend beyond that supplied by a single coronary artery. The most common wall motion abnormality described is the classical or typical pattern, as demonstrated in our case, with mid to apical left ventricular akinesia or dyskinesia. Other variants are inverted, mid left ventricular, global, biventricular, isolated right ventricular, and focal wall motion TTS.^4^ International takotsubo diagnostic criteria have been developed to improve diagnosis and stratification of TTS.^4^

Coronary angiography confirms the absence of culprit atherosclerotic coronary artery disease and intravascular ultrasound can rule out plaque rupture and intracoronary thrombosis.^4^ Cardiovascular magnetic resonance imaging is essential (Class IB indication)^1^ in the diagnostic workup of MINOCA to identify its precise cause and subsequent management and, where available, should be performed early, ideally during the inpatient admission.^1,5^ Furthermore, the international takotsubo diagnostic criteria recommend using CMR to exclude infectious myocarditis and to confirm the diagnosis of TTS.^4^

On CMR, positive EGE/LGE in TTS is not that frequently described in the literature, and absence of LGE was previously thought to be more indicative of TTS.^4^ In a study of 53 patients with TTS, 92% had early EGE.^6^ Early gadolinium enhancement–positive TTS patients had delayed recovery of regional wall motion abnormalities compared to EGE-negative TTS patients.^6^ Early gadolinium enhancement in TTS is likely due to hyperaemia and capillary leakage,^7^ or oedema.^8^

Late gadolinium enhancement can be present in the acute TTS setting in up to 41% of cases.^9^ In a case series of 23 TTS patients, Nakamori *et al.*^10^ found that 5 (22%) had LGE in the acute phase. Late gadolinium enhancement was transmural in all positive cases and had lower contrast-to-signal ratio when compared with infarcts.^10^ The LGE disappeared in all five patients after 12 months.^10^ Patchy mid-wall pattern,^11,12^ as was the case in our patient, and a well-defined transmural band at the junction between the dyskinetic segments and the hypercontractile segments are other patterns of LGE reported.^12^ Using electron microscopy and immunohistochemistry of endomyocardial biopsies, Rolf *et al.*^11^ proposed that LGE in TTS was due to a transient reactive fibrosis by demonstrating that extracellular collagen-1 content was higher in LGE-positive TTS patients compared to LGE-negative TTS patients, and that the LGE was not caused by cell necrosis or oedema. From the histopathologic analysis of cadaveric hearts in patients suffering from TTS, Maréchaux *et al.*^13^ demonstrated contraction band necrosis, which may be an alternative explanation for LGE that persists in TTS. Naruse *et al.*^14^ concluded that LGE-positive patients had a higher prevalence of cardiogenic shock, and their ECG and echocardiographic changes took longer to normalize compared to LGE-negative patients.

## Conclusion

Early CMR should be performed in MINOCA. Cardiovascular magnetic resonance imaging can provide high-quality illustrations of regional wall motion abnormalities, parametric T_1_/T_2_ mapping, and characteristic LGE patterns that may help confirm the diagnosis of TTS. Further research is required to identify the precise cause of the various patterns of EGE/LGE in TTS and their clinical significance.

## Supplementary Material

ytae016_Supplementary_Data

## Data Availability

The data underlying this article are available in the article and in its online Supplementary material.

## Lead author biography

Dr Ritesh Sunnasy is a cardiology imaging specialist registrar at the Essex Cardiothoracic Centre in Basildon, England. He completed his medical degree at the University of Birmingham, with an intercalated BSc at King’s College London. He recently completed a clinical fellowship in cardiovascular magnetic resonance imaging at the Royal Brompton Hospital. His specialist interests include CMR, cardiac CT, and echocardiography.

## Supplementary material

**Consent:** The patient has given written informed consent to the publication of their medical history, investigation, and management reports in accordance with COPE guidelines.

**Funding:** None declared.
