# Supplementary figures and images for: Parametric cardiovascular magnetic resonance imaging in takotsubo syndrome: a case report
Source: Eur Heart J Case Rep. 2024 Jan 8;8(1):ytae016. doi: 10.1093/ehjcr/ytae016 (PMC10903182; doi:10.1093/ehjcr/ytae016)

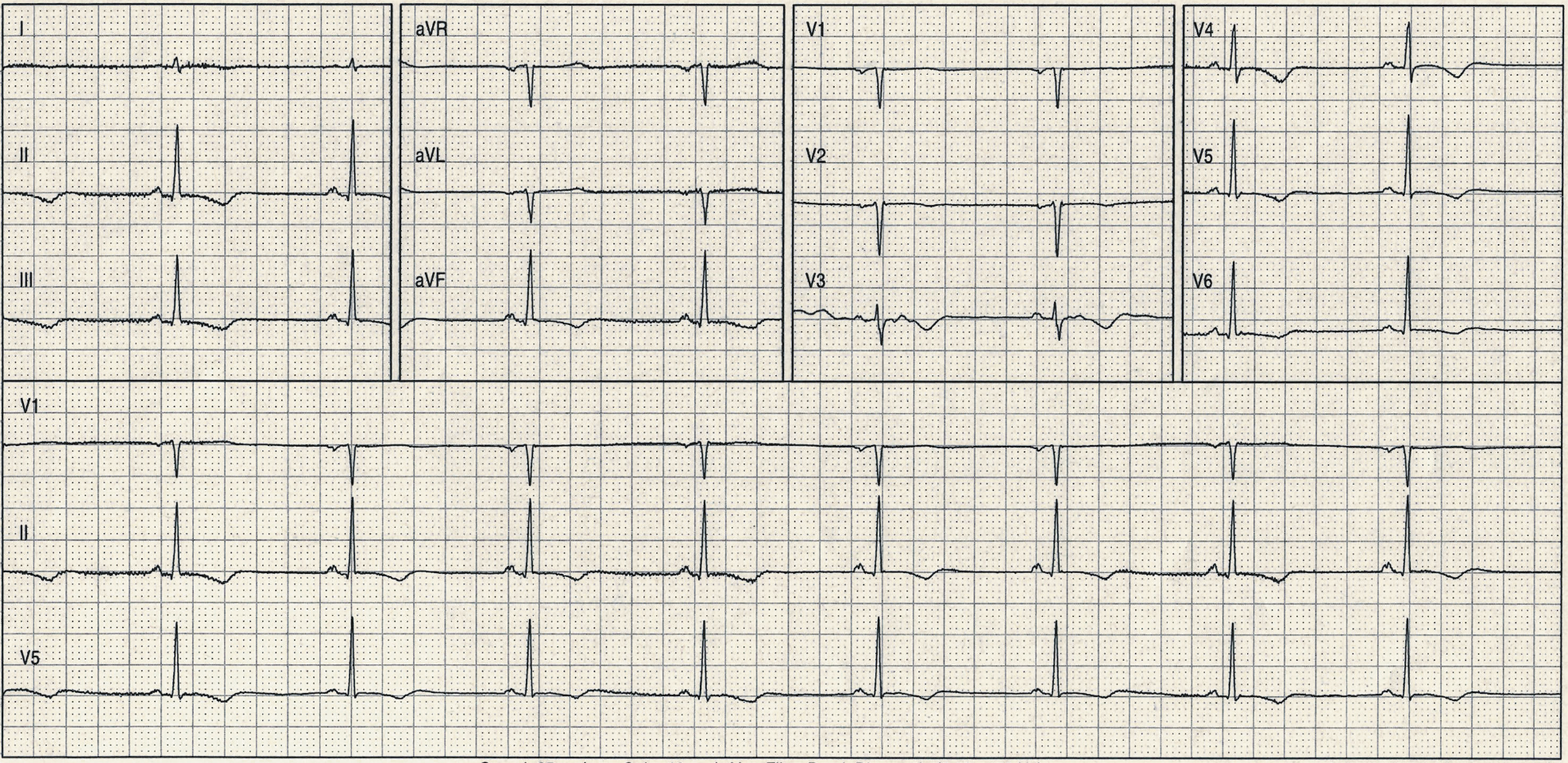

Supplement: ytae016_Supplementary_Data [file ytae016_Supplementary_Data.zip › Figure S1 - Discharge ECG.png]
